# Inhibitory Effects of Ginsenoside Compound K on Lipopolysaccharide-Stimulated Inflammatory Responses in Macrophages by Regulating Sirtuin 1 and Histone Deacetylase 4

**DOI:** 10.3390/nu15071626

**Published:** 2023-03-27

**Authors:** Hyunju Kang, Shin Kim, Jin-Young Lee, Bohkyung Kim

**Affiliations:** 1 Department of Food and Nutrition, Keimyung University, Daegu 42601, Republic of Korea; 2 Department of Immunology, School of Medicine, Keimyung University, Daegu 42601, Republic of Korea; 3 Department of Biological Sciences, Keimyung University, Daegu 42601, Republic of Korea; 4 Department of Food Science and Nutrition, Pusan National University, Busan 46241, Republic of Korea

**Keywords:** ginsenoside compound K, macrophage, inflammation, sirtuin 1, histone deacetylase 4

## Abstract

Inflammation, an innate immune response mediated by macrophages, has been a hallmark leading to the pathophysiology of diseases. In this study, we examined the inhibitory effects of ginsenoside compound K (CK) on lipopolysaccharide (LPS)-induced inflammation and metabolic alteration in RAW 264.7 macrophages by regulating sirtuin 1 (SIRT1) and histone deacetylase 4 (HDAC4). LPS suppressed SIRT1 while promoting HDAC4 expression, accompanied by increases in cellular reactive oxygen species accumulation and pro-inflammatory gene expression; however, the addition of CK elicited the opposite effects. CK ameliorated the LPS-induced increase in glycolytic genes and abrogated the LPS-altered genes engaged in the NAD+ salvage pathway. LPS decreased basal, maximal, and non-mitochondrial respiration, reducing ATP production and proton leak in macrophages, which were abolished by CK. SIRT1 inhibition augmented *Hdac4* expression along with increased LPS-induced inflammatory and glycolytic gene expression, while decreasing genes that regulate mitochondrial biogenesis; however, its activation resulted in the opposite effects. Inhibition of HDAC4 enhanced *Sirt1* expression and attenuated the LPS-induced inflammatory gene expression. In conclusion, CK exerted anti-inflammatory and antioxidant properties with the potential to counteract the alterations of energy metabolism, including glycolysis and mitochondrial respiration, through activating SIRT1 and repressing HDAC4 in LPS-stimulated macrophages.

## 1. Introduction

The pathogenesis of chronic diseases implicates inflammation and oxidative stress. Macrophages play an essential role in inflammatory responses, leading to chronic diseases [1,2]. They produce pro-inflammatory cytokines such as tumor necrosis factor α (TNFα) and interleukin-1β (IL-1β) and generate excessive reactive oxygen species (ROS) upon exposure to stimuli, including interferon γ or lipopolysaccharide (LPS) [3,4].

Ginseng has long been used in Asian countries as a traditional medicine for enhancing immunity and inhibiting inflammation and metabolic diseases [5]. The active components of ginseng are ginsenosides, which have a wide range of biological and pharmaceutical properties, including antioxidant and anti-inflammatory properties [6,7]. Ginsenoside compound K (CK) is a secondary metabolite biotransformed pharmacologically active protopanaxadiol (PPD)-type ginsenosides, with higher bioavailability and solubility than its parent ginsenosides [8]. CK has gained attention recently because of its antioxidant [9], anti-inflammatory [5,10], and anti-cancer properties [11]. The efficacy of CK has been extended to inhibit palmitate-induced skeletal muscle apoptosis by repressing endoplasmic reticulum stress [12] and to stimulate the secretion of glucagon-like peptide-1 (GLP1), a gastrointestinal hormone to ameliorate impaired glucose tolerance in type 2 diabetes db/db mice [13].

The beneficial effects of ginsenosides can be closely related to the histone modifications such as acetylation and deacetylation [11]. CK decreased histone deacetylase 1 (HDAC1) mRNA and protein expression, modulating histone H3 and H4 acetylation to inhibit growth and induce apoptosis in HT-29 human colon cancer cells [11]. Ginsenoside 20(s)-Rh2 suppressed HDAC1, HDAC2, and HDAC6 to inhibit the growth of human leukemia cells [14], and ginsenoside Rg3 decreased HDAC3 expression with concomitant inhibition of melanoma cell proliferation [15]. Activation of sirtuin 1 (SIRT1), a class III HDAC, by ginsenosides has emerged as a potential strategy for regulating inflammation, oxidative stress, and metabolic dysfunction [16,17,18]. SIRT1 is downregulated as part of the acute inflammatory response [19], whereas SIRT1 activation modulates inflammation and oxidative stress in LPS-activated macrophages [19]. Mounting evidence showed that ginsenosides increased NAD+ levels, a cofactor of SIRT1 [20], in metabolically active tissues, such as the liver, skeletal muscle, and brown adipose tissue, thus promoting mitochondrial biogenesis and oxidative phosphorylation thereby protecting against metabolic diseases [21,22].

Class IIa HDACs such as HDAC4 have been shown to activate toll-like receptors (TLRs) [23], leading to nuclear factor-κB (NF-κB) activation for pro-inflammatory gene expression [24,25]. Since the functions of HDACs are similar in that they catalyze the removal of acetyl groups from the lysine residues of both histone and non-histone proteins, a mechanistic link may exist between HDACs such as SIRT1 and HDAC4. In line with this notion, we previously demonstrated the potential crosstalk between SIRT1 and HDAC4 in regulating ethanol-induced inflammation in macrophages [26]. However, there has been little attention to the relationship between CK and histone deacetylases such as SIRT1 and HDAC4 in regulating inflammation and metabolic dysfunction in macrophages. In the present study, we explored whether CK activates SIRT1 in association with HDAC4 to inhibit inflammation, oxidative stress, and metabolic alterations in macrophages exposed to LPS. Furthermore, we determined the effects of CK on the mitochondrial biogenesis and NAD+ salvage pathway in LPS-stimulated macrophages.

## 2. Materials and Methods

### 2.1. Cell Culture and Treatment

Murine RAW 264.7 macrophages (ATCC, Manassas, VA, USA) were maintained in RPMI 1640 media (Gibco, Grand Island, NY, USA) with 10% fetal bovine serum (Welgene Inc., Daegu, South Korea), 100 U/mL penicillin, 100 μg/mL streptomycin, 1 x vitamins, and 2 mmol/L l-glutamine. Cells were incubated at 5% CO_2_ at 37 °C. CK, EX-527, a SIRT1 inhibitor, and resveratrol, a SIRT1 activator, were purchased from Sigma-Aldrich (St. Louis, MO, USA). Their stock solutions (10 mM) were made in dimethyl sulfoxide and kept at −80 °C until needed. The final concentrations of CK, EX-527, and resveratrol utilized in the experiments were 20 μM, 15 μM, and 20 μM, respectively. For analysis, after pretreatment with reagents for 3 h, cells were stimulated by 100 ng/mL LPS (Sigma-Aldrich) for the indicated time. Cell cytotoxicity using the trypan blue exclusion test was employed to determine the concentration of CK.

### 2.2. RNA Extraction and Real-Time Quantitative PCR (qRT-PCR)

According to the manufacturer’s instructions, total RNA was isolated from cells utilizing TRIzol reagent (Thermo Fisher Scientific, Waltham, MA, USA) Reverse transcription for cDNA synthesis was conducted using ReverTra Ace^®^ qPCR RT master mix (Code No. FSQ-201, Toyobo, Osaka, Japan). qRT-PCR analysis was conducted utilizing a LightCycler^®^ 480 Instrument II Real-Time PCR system (Roche Diagnostics, Basel, Switzerland) or Bio-Rad CFX96 Touch Real-Time PCR system (Bio-Rad, Hercules, CA, USA). Three independent experiments were performed. Primers were designed using Beacon Designer 7 software (PREMIER Biosoft International, Palo Alto, CA, USA) according to the GenBank database.

### 2.3. Western Blot Analysis

Total cell lysates were prepared and Western blot analysis was conducted as described [27]. Antibodies for SIRT1, hexokinase 1 (HK1), and glyceraldehyde 3-phosphate dehydrogenase (GAPDH) were from Santa Cruz Biotechnology (Santa Cruz, CA, USA). Antibodies for HDAC4 and glucose transporter 1 (GLUT1) were obtained from Cell Signaling Technology (Danvers, MA, USA). GAPDH was employed as a loading control to normalize data in the total protein. A Chemi Image documentation system (Fusion FX7, Vilber Lourmat, Collégien, France) or Chemidoc XRS+ system (Bio-Rad) was used to image the blots. Independent experiments were performed at least three times.

### 2.4. Measurements of Cellular ROS Levels

As previously mentioned [28], 2′,7′-dichlorofluorescein (DCFH; Sigma-Aldrich) was utilized to measure cellular ROS levels. At least three repeats of experiments were performed independently.

### 2.5. Small Interfering RNA (siRNA) Transfection

RAW 264.7 macrophages were transfected with control siRNA (Santa Cruz Biotechnology) or *Hdac4* siRNA (Thermo Fisher Scientific). Transfections were carried out with Lipofectamine RNAiMAX (Thermo Fisher Scientific). Cells were treated with 100 ng/mL LPS for 16 h following the transfection for subsequent gene analysis. At least two independent experiments were performed.

### 2.6. Energy Metabolism of Cells

After RAW 264.7 cells were pre-treated with CK for 3 h and activated by 100 ng/mL LPS for 3 h, cells were applied to an Extracellular Flux Analyzer (Seahorse Bioscience, North Billerica, MA, USA) for a Mito Stress test. Total DNA was extracted from each well after assay using a Clear-S™ Quick DNA Extraction Kit (Invirustech, Gwangju, South Korea) to normalize the data. At least two independent repeats of the experiments were performed.

### 2.7. Statistical Analysis

To determine significant differences between groups, one-way analysis of variance (ANOVA) and the Newman–Keuls post hoc test, two-way ANOVA, and Tukey’s post hoc test or unpaired *t*-test were performed. In order to analyze the data, GraphPad Prism 9.0 (GraphPad Software, La Jolla, CA, USA) was used. The data are shown as mean ± SD. A *p*-value of 0.05 or less was regarded as significant.

## 3. Results

### 3.1. CK Supressed the LPS-Induced Inflammation and ROS Generation in Macrophages

**Figure 1:**
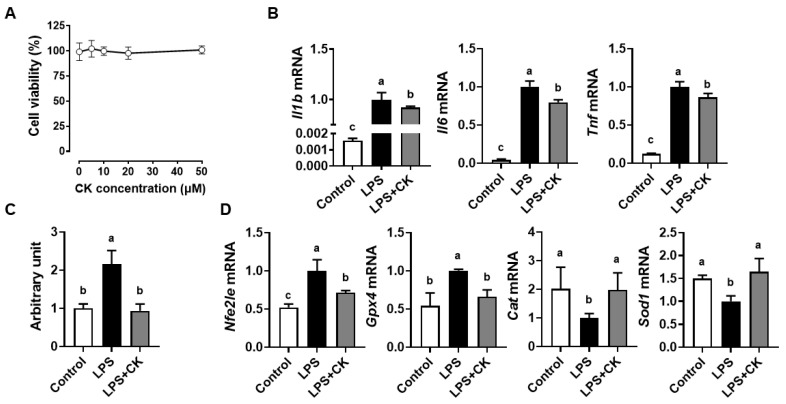
CK inhibits LPS-induced inflammation and ROS accumulation in macrophages. RAW 264.7 macrophages were treated with increasing CK concentrations from 0 to 50 µM for 24 h for cell cytotoxicity (**A**). Following a 3 h pre-treatment with 20 μM CK, RAW 264.7 macrophages were activated with or without 100 ng/mL of LPS for 16 h for pro-inflammatory gene analysis (**B**), ROS accumulation (**C**), and antioxidant gene expression (**D**). Mean ± SD. Bars without sharing a common letter are significantly different (*p* < 0.05). CK, ginsenoside compound K.

To investigate the cytotoxicity of CK, increasing doses of CK were administered to RAW 264.7 macrophages. The viability of macrophages treated with CK up to 50 μM showed no significant differences. Thus, the subsequent experiments were carried out with a concentration of 20 μM of CK (Figure 1A). LPS-activated macrophages induce pro-inflammatory cytokine production [3,4]. The LPS exposure enhanced mRNA levels of *Il1b*, *Il6*, and *Tnf*, which were abolished by CK in RAW 264.7 cells (Figure 1B), indicating that CK has an anti-inflammatory impact on macrophages activated by LPS. Cellular ROS levels were markedly induced by LPS, but CK diminished the increase to a baseline level (Figure 1C). The oxidative microenvironment driven by LPS treatment forces macrophages to produce the antioxidant genes to avoid oxidative damage [3]. The mRNA levels of nuclear factor E2-related factor 2 (*Nfe2l2* or *Nrf2*) and glutathione peroxidase 4 (*Gpx4*) were significantly increased, whereas LPS decreased catalase (*Cat*) and superoxide dismutase 1 (*Sod1*) in RAW 264.7 macrophages (Figure 1D). However, CK significantly attenuated the LPS-induced alteration, demonstrating the antioxidant efficacy of CK. 

### 3.2. CK and Hdac4 Knockdown Abolished the LPS-Induced Reduction in Sirt1 and Induction of Pro-Inflammatory Gene Expression in Macrophages

**Figure 2:**
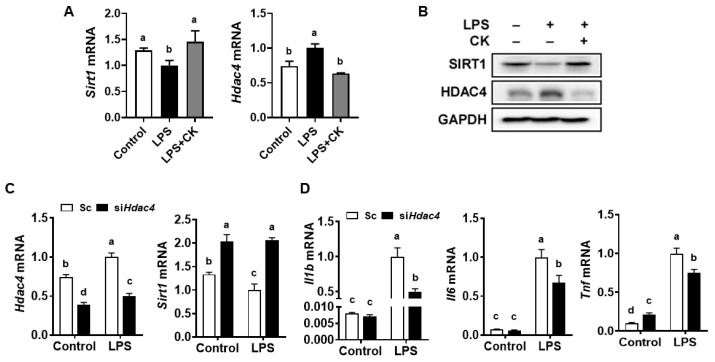
CK and *Hdac4* knockdown abolish decreased SIRT1 and increased pro-inflammatory gene expression by LPS in macrophages. Following a 3 h pre-treatment with 20 μM CK, RAW 264.7 macrophages were stimulated with or without 100 ng/mL of LPS for 16 h for gene analysis (**A**). RAW 264.7 macrophages were treated as previously mentioned for Western blot analysis. A representative blot image is displayed (**B**). Scrambled control (Sc) and siRNA against *Hdac4* (si*Hdac4*) were transfected into RAW 264.7 macrophages for 24 h, after which they were treated with or without 100 ng/mL of LPS for 16 h for gene analysis (**C**,**D**). Mean ± SD. Bars without sharing a common letter are significantly different (*p* < 0.05). CK, ginsenoside compound K.

SIRT1 is a crucial regulator of inflammation by suppressing the release of pro-inflammatory cytokines and ROS accumulation in macrophages [29,30]. LPS has been reported to decrease *Sirt1* expression in RAW 264.7 macrophages and murine preosteoblastic MC3T3-E1 cells [31,32]. The expression of HDAC4 increased in response to the stimulation of macrophages with the activation of TLRs and NF-κB [24,25]. Therefore, we next determined the impact of CK on SIRT1 and HDAC4 expression in LPS-activated macrophages. LPS decreased *Sirt1* but elevated *Hdac4* mRNA levels, which was ameliorated by CK in RAW 264.7 cells (Figure 2A). Consistently, reduced SIRT1 and increased HDAC4 protein by LPS were restored by CK in macrophages (Figure 2B), implying a relationship between SIRT1 and HDAC4, given the effects of CK on the macrophages’ response to LPS stimulation.

Next, we knocked down *Hdac4* using siRNA to investigate the effects of HDAC4 on *Sirt1* and pro-inflammatory gene expression in macrophages. Knockdown of *Hdac4* increased *Sirt1* expression in comparison to the scrambled control, both without and with LPS exposure (Figure 2C), exhibiting the inhibitory effect of HDAC4 on the expression of SIRT1. In addition, *Hdac4* deficiency decreased the LPS-induced expression of *Il1b*, *Il6*, and *Tnf* compared to the scrambled control in LPS-stimulated macrophages (Figure 2D).

### 3.3. Regulation of SIRT1 Activity Regulated LPS-Induced Alterations in Hdac4 and Pro-Inflammatory Gene Expression in Macrophages

**Figure 3:**
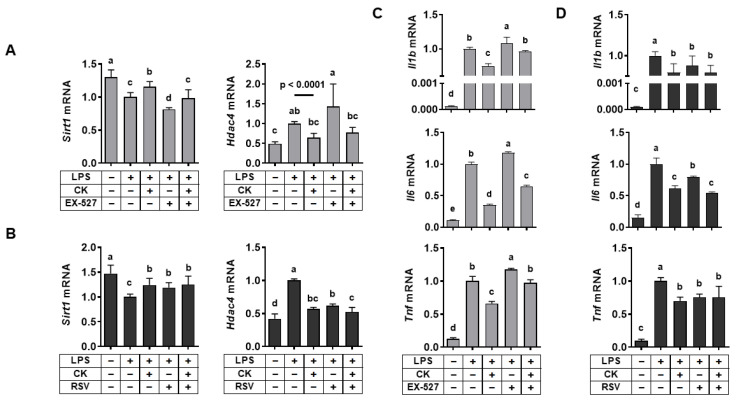
Regulation of SIRT1 activity affects LPS-induced *Hdac4* and pro-inflammatory gene expression in macrophages. Following a 3 h pre-treatment with 20 μM CK, RAW 264.7 macrophages were stimulated with or without 100 ng/mL of LPS for 16 h in the absence or presence of 15 μM EX-527 (**A**,**C**) or 20 μM resveratrol (**B**,**D**) to analyze genes. Mean ± SD. Bars without sharing a common letter are significantly different (*p* < 0.05). CK, ginsenoside compound K.

The efficacy of ginsenosides in reducing inflammation is reportedly due to the SIRT1 signaling pathway [16,17,18]. Thus, we examined whether the protective effects of CK were ascribed to the role of SIRT1, since CK is a metabolite of ginsenosides. We used EX-527, a SIRT1 inhibitor [33,34], and resveratrol, a SIRT1 activator [35,36], respectively, to determine the role of CK in regulating *Sirt1* and *Hdac4* expression. EX-527 augmented the LPS-induced decrease in *Sirt1* and increased *Hdac4* levels in RAW 264.7 macrophages, which were abolished by CK (Figure 3A). However, the LPS-induced reduction of *Sirt1* and induction of *Hdac4* expression were inhibited by resveratrol, supporting the CK function (Figure 3B).

Next, in macrophages exposed to LPS, the role of SIRT1 to the anti-inflammatory impact of CK was assessed. LPS-increased pro-inflammatory gene expression (*Il1b*, *Il6*, and *Tnf*) was further induced by EX-527, which was attenuated by CK in macrophages (Figure 3C). However, increased *Il1b*, *Il6*, and *Tnf* mRNA levels by LPS were significantly abrogated by CK or resveratrol alone (Figure 3D). In the combination of CK and resveratrol, there was a further reduction in *Il6* mRNA levels in LPS-stimulated macrophages. The results demonstrated the efficacy of CK in inhibiting LPS-increased pro-inflammatory gene expression might be ascribed to the role of SIRT1. In addition, the interaction between SIRT1 and HDAC4 may be involved in repressing the LPS-triggered pro-inflammatory gene expression by CK. Consistently, we previously showed crosstalk between SIRT1 and HDAC4 in regulating inflammation and oxidative stress induced by ethanol in macrophages [26].

### 3.4. Regulation of SIRT1 Activity Modulated Gene Expression Associated with the NAD+ Salvage Pathway in LPS-Stimulated Macrophages

**Figure 4:**
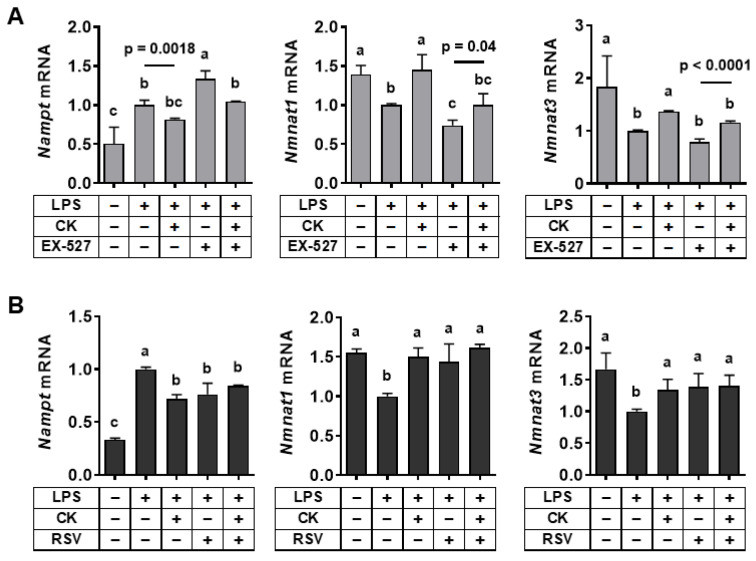
Regulation of SIRT1 activity affects the expression of genes related to the NAD+ salvage pathway in LPS-stimulated macrophages. Following a 3 h pre-treatment with 20 μM CK, RAW 264.7 macrophages were stimulated with or without 100 ng/mL of LPS for 16 h in the absence or presence of 15 μM EX-527 (**A**) or 20 μM resveratrol (**B**) to analyze genes. Mean ± SD. Bars without sharing a common letter are significantly different (*p* < 0.05). CK, ginsenoside compound K.

Recent advances have highlighted the pivotal role of NAD+ generation in the programming of macrophage function in the inflammatory response [37]. The expression of nicotinamide phosphoribosyltransferase (NAMPT), a key enzyme in the NAD+ salvage pathway, is known to alter macrophages’ inflammatory potential, as NAD+ depletion and increased NAMPT expression occurred rapidly after inflammatory activation [38,39]. To evaluate the role of CK in the NAD+ salvage pathway, we measured the expression of enzymes involved in the NAD+ salvage pathway. NAD+ hydrolysis by NAD+-consuming enzymes, such as SIRTs, results in the production of nicotinamide (NAM), which is converted to nicotinamide mononucleotide (NMN) by NAMPT. Then, nicotinamide mononucleotide adenylyltransferase 1 (NMNAT1) and NMNAT3 convert NMN to NAD+ in the nucleus and mitochondria, respectively [40]. While LPS increased *Nampt* expression, mRNA levels of *Nmnat1* and *Nmnat3* were decreased by LPS, which was abolished by CK (Figure 4). We examined whether the efficacy of CK on the NAD+ salvage pathway is ascribed to the role of SIRT1. When EX-527 inhibited SIRT1, the expression of *Nampt* was enhanced but mRNA levels *Nmnat1* and *Nmnat3* were reduced in LPS-stimulated macrophages, which was reversed by CK (Figure 4A). In contrast, LPS-induced alteration in *Nampt*, *Nmnat1*, and *Nmnat3* mRNA levels was abolished when SIRT1 was activated by resveratrol (Figure 4B). These findings demonstrated that SIRT1 might contribute to the efficacy of CK to support the NAD+ salvage pathway in LPS-exposed macrophages.

### 3.5. CK Ameliorated the LPS-Induced Glycolytic Capacity of Macrophages through SIRT1 Activation

**Figure 5:**
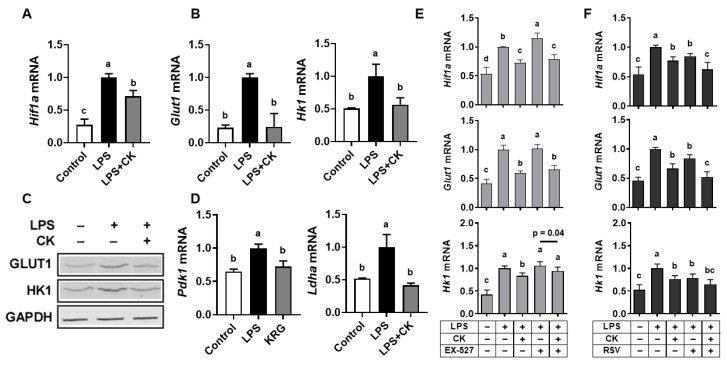
CK attenuates the LPS-induced glycolytic capacity in macrophages through SIRT1 activation. Following a 3 h pre-treatment with 20 μM CK, RAW 264.7 macrophages were stimulated with or without 100 ng/mL of LPS for 16 h for gene analysis (**A**,**B**,**D**) and protein measurement via Western blot (**C**). A representative blot image is displayed for Western blot analysis. Following a 3 h pre-treatment with 20 μM CK, cells were stimulated with or without 100 ng/mL of LPS for 16 h in the absence or presence of 15 μM EX-527 (**E**) or 20 μM resveratrol (**F**) to analyze genes. Mean ± SD. Bars without sharing a common letter are significantly different (*p* < 0.05). CK, ginsenoside compound K.

Metabolic pathways for energy production are known to be closely associated with the immune system, and macrophages can switch metabolic phenotypes in response to extracellular stimuli [25,41]. In particular, the Warburg effect, an increased glucose uptake rate and preferential production of lactate, even in the presence of oxygen, is critical for activating macrophages in response to LPS [42]. Thus, we determined the potential function of CK in modulating glycolysis in macrophages exposed to LPS. The major cell membrane glucose transporter in macrophages, GLUT1, is regulated by hypoxia-inducible factor 1α (HIF-1α) [43]. The entrance of glucose into macrophages requires HK1, as HK1 phosphorylates glucose to glucose-6-phosphate. LPS significantly induced mRNA levels of *Hif1a*, *Glut1*, and *Hk1*, but CK nullified the induction (Figure 5A,B). Consistent with mRNA expression, LPS increased GLUT1 and HK1 protein levels, but CK inhibited the increases close to the basal levels (Figure 5C).

The end-product of glycolysis, pyruvate, enters the mitochondria and is converted into acetyl-CoA by the pyruvate dehydrogenase (PDH) complex, whose activity is inhibited by PDH kinase (PDK) [44,45]. Instead, lactate dehydrogenase (LDH) converts pyruvate to lactate in the cytosol [44,45]. The effect of CK on these enzymes that control pyruvate’s metabolic fate was examined. The expression of *Pdk1* and *Ldha* was significantly induced by LPS and nullified by CK (Figure 5D), suggesting that LPS shifted energy metabolism to increase glycolysis, which was abolished by CK.

Next, we examined whether the efficacy of CK on glucose metabolism is attributed to the role of SIRT1 in LPS-stimulated macrophages. Inhibition of SIRT1 by EX-527 markedly increased the expression of genes associated with glycolysis, which was abolished by CK (Figure 5E); however, activation of SIRT1 by resveratrol or the combination of CK and resveratrol showed the opposite effects (Figure 5F). The results indicate that the role of CK in inhibiting LPS-altered glucose metabolism is at least in part responsible for SIRT1 activation in macrophages.

### 3.6. CK Regulated the LPS-Altered Mitochondrial Respiration in Macrophages

**Figure 6:**
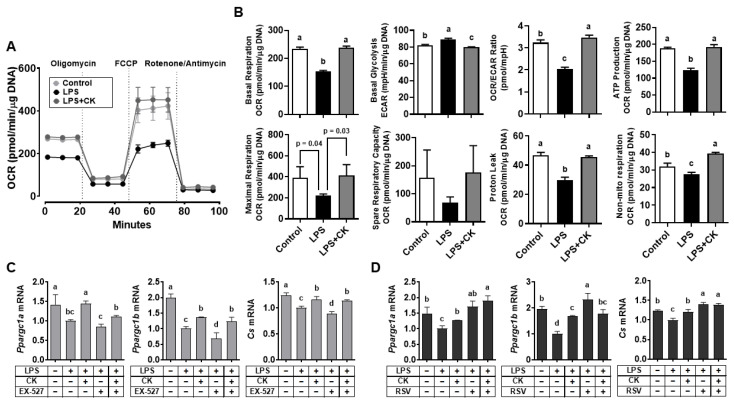
CK regulates the LPS-induced alteration in mitochondrial respiration in RAW 264.7 macrophages. Following a 3 h pre-treatment with 20 μM CK, cells were stimulated with or without 100 ng/mL of LPS for Mito Stress tests (**A**,**B**). Following a 3 h pre-treatment with 20 μM CK, cells were stimulated with or without 100 ng/mL of LPS for 16 h in the absence or presence of 15 μM EX-527 (**C**) or 10 μM resveratrol (**D**) to analyze genes. Mean ± SD. Bars without sharing a common letter are significantly different (*p* < 0.05). CK, ginsenoside compound K.

As mitochondria play a crucial role in cellular respiration and energy production [46], we further considered the efficacy of CK on mitochondrial respiration in LPS-stimulated RAW 264.7 macrophages. LPS treatment decreased basal respiration, ATP production, and proton leak, with a concomitant decrease in maximal respiration and non-mitochondrial respiration; however, LPS increased basal glycolysis in macrophages (Figure 6A,B). CK recovered the LPS-induced changes in mitochondrial respiration, repressing glycolysis.

LPS also significantly reduced the expression of peroxisome proliferator-activated receptor gamma coactivator 1-α (*Ppargc1a*) and *Ppargc1b*, important activators of mitochondrial biogenesis [47,48]. Citrate synthase (Cs), an enzyme that controls the rate for catalyzing the first step of the TCA cycle and biomarker of mitochondrial content, regulates the mitochondrial respiratory chain [46,49]. To examine whether the efficacy of CK depends on SIRT1 activation in mitochondrial respiration, the effects of the SIRT1 inhibitor and activator on the expression of genes engaged in mitochondrial biogenesis were examined. Interestingly, mRNA levels of *Ppargc1a*, *Ppargc1b*, and *Cs* were further reduced by EX-527 but elevated by resveratrol or CK, respectively, in LPS-stimulated macrophages (Figure 6C,D), demonstrating that the efficacy of CK is likely due to the role of SIRT1.

## 4. Discussion

Various functions of CK have been demonstrated to abolish inflammation, oxidative stress, and metabolic dysfunctions [5,10]. However, information regarding the effects of CK on SIRT1 has been obscure, although ginseng has been known to activate SIRT1 [16,17,18]. Since CK is a metabolite of ginseng, we speculated that SIRT1 might contribute to the protective efficacy of CK in LPS-treated macrophages. Among various ginsenosides, Rb1 can be transformed into CK by gut microbiota in the form of PPD type, which can bind strongly to SIRT1 [50]. Thus, the chemical structure of CK may be suitable for its relatively higher SIRT1 activity. In the present study, we found that CK activated SIRT1 in repressing LPS-induced inflammation, oxidative stress, and alterations in glycolysis and mitochondrial respiration.

SIRT1 has been reported to repress the transcriptional activity of NF-κB by directly deacetylating the RelA/p65 protein [51,52]. We also previously showed that SIRT1 activation suppressed ethanol-induced inflammation by abrogating the nuclear translocation of NF-κB p65 in macrophages [26,53]. The activation of SIRT1 repressed LPS-induced inflammation in alveolar macrophages [54]. CK has been reported to attenuate amyloid β-induced neuroinflammation in microglia BV2 cells by suppressing NF-κB p65 nuclear translocation [10]. We consistently found that CK repressed inflammation and ROS accumulation and restored decreased SIRT1 in LPS-stimulated macrophages. Moreover, inhibition or activation of SIRT1 resulted in the corresponding decrease or increase in CK efficacy on pro-inflammatory gene expression, respectively.

Significant increases in ROS due to LPS stimulation activates NRF2, which is bound to the repressor Kelch-like ECH-associated protein 1 (KEAP1) [55]. ROS bind to the cysteine residue of KEAP1, allow NRF2 to translocate to the nucleus, and bind to the antioxidant response element (ARE), inducing transcription of ARE-dependent antioxidant genes [55]. We found that CK attenuated the increased *Nfe2le* expression by LPS. NRF2 neutralizes ROS to protect cells from oxidative stress by inducing antioxidant enzymes such as *Cat*, *Gpx4*, and *Sod1*. Ginsenoside Rd, one of the CK precursors [56], ameliorated auditory cortex injury by SIRT1 activation, alleviating oxidative damage in guinea pigs [57]. Considering that SIRT1 is associated with NRF2 under oxidative conditions [50], these results indicate that the efficacy of CK in suppressing inflammation and oxidative stress in LPS-stimulated macrophages is ascribed to SIRT1’s function.

NAD+ plays an essential role in maintaining the antioxidant defense system and the electron transport chain [58]. LPS-activated macrophages may mediate the NAD+ salvage pathway to support cellular NAD+ levels by immune programming functions in the inflammatory response [37]. NAM, generated from the NAD+ hydrolysis through NAD+-consuming enzymes such as SIRT1, is recycled to NMN by NAMPT and then converted to NAD+ by NMNAT 1 and 3, constituting the NAD+ salvage pathway [40,59]. NAMPT promotes NAD+ production and maintains mitochondrial contents under oxidative stress to support mitochondrial NAD+ levels [60]. Furthermore, NMN supplementation attenuates oxidative stress in aged cerebral microvascular endothelial cells and promotes SIRT1 activation and anti-inflammatory effects [61].

Notably, LPS treatment increased *Nampt* levels but decreased the levels of *Nmnat1* and *Nmnat3*, leading to a less effective NAD+ salvage pathway. To compensate for the lack of NAD+ in LPS-stimulated macrophages, CK increased the expression of genes involved in the NAD+ salvage pathway. Ginsenoside Rb1, which can be converted to CK [56], has been reported to be crucial against hyperglycemic oxidative damage by modulating the NAD+-SIRT1 axis [62]. NAD+, a cofactor that activates SIRT1 [63], can also be supported by CK. Given the information, SIRT1 activation may contribute to CK’s efficacy in inducing the NAD+ salvage pathway in inflammatory macrophages. Taken together, CK may counteract the LPS-induced reduction of NAD+ levels by facilitating the salvage pathway in macrophages.

Interaction between SIRT1 and HDAC4 was encountered in regulating the inflammation of macrophages, since SIRT1 activation by resveratrol has been reported to translocate HDAC4 from the nucleus to the cytoplasm, thereby inhibiting gluconeogenesis in insulin-resistant hepatocytes [64]. Upregulation of *Hdac4* resulted in the downregulation of *Sirt1* in activated mouse hepatic stellate cells [65] and skeletal muscle cells stimulated with interferon γ [66]. Knockdown of *Hdac4* inhibited TNF-induced ROS production and transcriptional activity of NF-κB in cultured rat mesenteric arterial smooth muscle cells [67]. Previously, we also demonstrated that inhibition of *Hdac4* enhanced *Sirt1* expression with a concomitant reduction in pro-inflammatory genes, but its overexpression elicited the opposite consequences in ethanol-exposed macrophages [26]. Furthermore, we showed that loss of *Hdac4* in macrophages exacerbated inflammation of liver and adipose tissue in male mice with diet-induced non-alcoholic steatohepatitis [68]. This evidence suggests that HDAC4 likely exerts pro-inflammatory functions via NF-κB activation. Similarly, in this study, inhibition of SIRT1 increased *Hdac4* expression, accompanied by increased pro-inflammatory gene expression, but activation of it showed the opposite effects in LPS-treated macrophages. Here, CK was shown to increase SIRT1 but decrease HDAC4 expression in LPS-stimulated macrophages. In addition, inhibition of *Hdac4* resulted in increased *Sirt1* expression, abrogating the LPS-induced pro-inflammatory gene expression. These findings indicate an interaction between SIRT1 and HDAC4 by employing CK to suppress inflammation and oxidative stress induced by LPS in macrophages. When inflammation is triggered, HDAC4 activates TLRs [23]; however, SIRT1 downregulates TLR4 to inhibit inflammation through deacetylation of NF-κB p65 [69]. HDAC4 suppressed SIRT1 by recruiting HDAC4 to the *Sirt1* promoter to repress its transcription [65,66]. The interaction between SIRT1 and HDAC4 may result from competition for binding to the Sp1 transcription factor for their transcription [70,71].

Energy metabolism depends on the balance of macrophage phenotypes. The polarization of macrophages induces metabolic reprogramming, such as increased glycolysis in the pro-inflammatory phenotype but mitochondrial oxidative phosphorylation in the anti-inflammatory phenotype as a major energy source [72,73]. We found that LPS increased pro-inflammatory and glycolytic gene expression, indicating increased glycolysis, which was abolished by CK. The metabolic shift toward aerobic glycolysis by inflammatory macrophages [4] may be counteracted by CK via changes in macrophage energy metabolism. Notably, in the current study, inhibition of SIRT1 using EX-527 further increased the expression of glycolytic genes, whereas activation of SIRT1 by resveratrol repressed them in LPS-stimulated macrophages. The results suggested that SIRT1 may contribute to the efficacy of CK in inhibiting glycolysis. The HIF-1α protein can be modified at the posttranslational level by reversible lysine acetylation at the two different acetylation sites, such as residues Lys-532 and Lys-674 [74,75]. The acetylation at Lys-532 induces von Hippel-Lindau (VHL)-dependent HIF-1α protein degradation [74], while acetylation at Lys-674 activates HIF-1α transcriptional activity [75]. Interestingly, the deacetylation at Lys-674 is mediated by SIRT1 [75], but deacetylation at Lys-532 can be mediated by HDAC4 [74]. HDAC4 was reported to promote HIF-1α transactivation by increasing binding to transcriptional co-activator, p300 [76]. Thus, we speculated that LPS increased pro-inflammatory gene expression and activated HDAC4 to deacetylate Lys-532 and activate HIF-1α, increasing glycolysis in macrophages. However, CK activated SIRT1, which deacetylates Lys-674 to repress HIF-1α activity and inhibited HDAC4, reducing glycolysis. Increased HIF-1α with increased target genes, including *Glut1* and *Hk1*, indicate increased glucose entry into macrophages. Inhibition of PDH activity by PDK decreased histone H3 acetylation, converting pyruvate to lactate under prolonged hypoxic conditions [77]. Lactate induces PDK1 expression, enhancing the HIF-1α signaling and upregulating glycolysis while downregulating the TCA cycle [78]. Given that CK decreases *Pdk1* and *Ldha* expression, CK inhibits glycolysis by decreasing glucose entry and the rate of glycolysis in macrophages. These findings imply that the efficacy of CK in restoring the LPS-induced energy metabolic shift may be partly due to SIRT1 activation [75] and HDAC4 inhibition in macrophages [26].

LPS-induced disruption of redox balance promotes an inflammatory response in mitochondria with increased glycolysis [79,80]. LPS-induced reduction in basal, maximal, and non-mitochondrial respiration with decreased ATP production and proton leak indicates mitochondrial dysfunction, which led to increased glycolysis for energy production. We found that CK recovered the basal oxygen consumption rate (OCR), with concomitant repression of the basal extracellular acidification rate (ECAR), indicating that CK may switch the energy metabolism from glycolysis to oxidative phosphorylation, increasing ATP production. CK also restored the decreased *Ppargc1a*, *Ppargc1b*, and *Cs* by LPS, implying its ability to regulate mitochondrial energy metabolism [47,48]. Interestingly, mRNA levels of *Ppargc1a*, *Ppargc1b*, and *Cs* were reduced by EX-527, but increased by resveratrol, respectively, demonstrating the dependence of CK on the role of SIRT1. SIRT1 controls mitochondrial biogenesis in hepatocytes by deacetylating PGC-1α in an NAD+-dependent manner [81] and regulates glucose metabolism by forming the SIRT1-PGC-1α axis [82]. Both PGC-1α and PGC-1β were reported to inhibit inflammation, allowing macrophages to be activated alternatively [48,81]. Previously, we also demonstrated that nicotinamide riboside, an NAD+ precursor, could restore the mitochondrial energy metabolism through SIRT1 activation by normalizing NAD+/NADH ratios in ethanol-induced macrophages [83]. Taken together, the regulatory effect of CK on the metabolic switch may be attributed to SIRT1 activation, in addition to the induction of mitochondrial biogenesis-related gene expression in LPS-treated macrophages.

## 5. Conclusions

In summary, this study demonstrated that the crosstalk between SIRT1 and HDAC4 may contribute to the properties of CK exhibiting anti-inflammatory and antioxidant effects in LPS-stimulated macrophages. CK abolished the LPS-induced glycolysis and mitochondrial dysfunction regulating the NAD+ salvage pathway in macrophages by activating SIRT1 while repressing HDAC4. The present study provided new information about CK’s ability to activate SIRT1 and mechanistic understanding of SIRT1 and HDAC4 crosstalk in suppressing inflammation in macrophages. The findings may provide therapeutic strategies for inflammation and metabolic disorders using CK.

## Data Availability

All data generated or analyzed during this study are included in the published article.
